# Hand eczema and other inflammatory skin diseases: efficacy of oral alitretinoin

**DOI:** 10.1186/2045-7022-5-S1-O23

**Published:** 2015-03-11

**Authors:** Elisa Boni, Stefano Pattini, Mario Guanti, Francesca Giusti, Giovanni Pellacani, Patrizia Pepe

**Affiliations:** 1 Azienda Ospedaliero-Universitaria Policlinico Di Modena, Dermatology Department, Allergy Unit, Modena; Italy; 2 Azienda Ospedaliero-Universitaria Policlinico De Modena, Dermatology Department, Allergy Unit, Modena; Italy; 3 Azienda Ospedaliero-Universitaria Policlinico di Modena, Dermatology Department, Modena; Italy; 4 Azienda Ospedaliero-Universitario Policlinico Di Modena, Dermatology Department, Modena, Italy

## Background

Alitretinoin is a vitamin A derivative. It’s a pan-agonist of Retinoic Acid Receptors and Retinoid X Receptors. It interferes with keratinocytes’ proliferation and differentiation and exerts immunomodulatory and anti-inflammatory activity. Its clinical efficacy in the treatment of chronic hand eczema is well demonstrated. Recently, various case reports and case series pointed out its efficacy in other skin inflammatory disorders such as atopic dermatitis, psoriasis, lichen planus, Darier’s disease, Hailey Hailey’s disease, lichen simplex, pityriasis rubra pilaris and cutaneous lupus erythematosus.

## Patients and method

We assessed the efficacy of oral alitretinoin in hand eczema and in other inflammatory skin disorders with palmar and extra-palmar localization. In the last 2 years, 41 patients received oral alitretinoin for the treatment of severe chronic hand eczema. 24 of them exclusively suffered of eczema with palmar localization. On the other side, 17 showed different clinical manifestations involving hands and other skin areas: nine with palmo-plantar eczema; two with eczema of the hands, feet and limbs; one had widespread hyperkeratotic eczema; one patient with severe atopic dermatitis and another one with nummular eczema; three patients suffered of psoriasis of the feet and/or hands.

## Results

36 patients received treatment for at least 3 months and up to 9 month. In 72.2% of them, remission of hand eczema was observed, evaluated as clear or almost clear hands according to “Physician Global Assessment” score. The median time to relapse was 6.4 months after discontinuation of treatment. Clinical response or improvement was also observed in patients suffering of eczema of the feet, widespread eczema, atopic dermatitis and psoriasis.

## Conclusion

This study provides evidence that oral alitretinoin is effective in hand eczema. We confirm oral alitretinoin as desirable treatment in chronic hand eczema due to its anti-inflammatory activity and safety profile without altering skin barrier function. Moreover, its shorter half-life makes it a preferable treatment in women in childbearing age compared with other retinoids. Our data also showed its efficacy in other skin inflammatory diseases, as previously reported in the literature. Further studies will have to support its use in other skin inflammatory diseases with palmar and extra-palmar localization.

